# Protective Role of the Nucleic Acid Sensor STING in Pulmonary Fibrosis

**DOI:** 10.3389/fimmu.2020.588799

**Published:** 2021-01-08

**Authors:** Florence Savigny, Corinne Schricke, Norinne Lacerda-Queiroz, Mélanie Meda, Mégane Nascimento, Sarah Huot-Marchand, Felipe Da Gama Monteiro, Bernhard Ryffel, Aurélie Gombault, Marc Le Bert, Isabelle Couillin, Nicolas Riteau

**Affiliations:** Experimental and Molecular Immunology and Neurogenetics Laboratory (INEM), CNRS Orleans (UMR7355) and University of Orleans, Orleans, France

**Keywords:** idiopathic pulmonary fibrosis, STING, self-DNA recognition, mice, IL-28

## Abstract

Idiopathic pulmonary fibrosis (IPF) is the most common and severe type of interstitial lung disease for which current treatments display limited efficacy. IPF is largely driven by host-derived danger signals released upon recurrent local tissue damage. Here we explored the roles of self-DNA and stimulator of interferon genes (STING), a protein belonging to an intracellular DNA sensing pathway that leads to type I and/or type III interferon (IFN) production upon activation. Using a mouse model of IPF, we report that STING deficiency leads to exacerbated pulmonary fibrosis with increased collagen deposition in the lungs and excessive remodeling factors expression. We further show that STING-mediated protection does not rely on type I IFN signaling nor on IL-17A or TGF-β modulation but is associated with dysregulated neutrophils. Together, our data support an unprecedented immunoregulatory function of STING in lung fibrosis.

## Introduction

Idiopathic pulmonary fibrosis (IPF) is the most common type of idiopathic interstitial pneumonia (1, 2), characterized by progressive lung scarring causing shorter life expectancy and high mortality rate (2, 3). While the etiology is still unclear, it is believed that the physiopathology relies on repeated local micro-injuries triggering DNA damage, unbalanced cell death and aberrant tissue remodeling with extracellular matrix components and ultimately fibrosis (1–4). Immune cells such as macrophages, neutrophils and T helper 17 (Th17) cells recruited to the lung tissue are known to display important proinflammatory and profibrotic functions (5). Self-derived danger-associated molecular patterns (DAMPS) are considered as important contributors (6), such as ATP (7) and uric acid (8) to the pathology. In this study, we hypothesize that self-nucleic acid sensing might also contribute to IPF. Pathogen-derived nucleic acid sensing through pathogen recognition receptors (PRRs) is an effective strategy to detect invading microorganisms and trigger innate and adaptive immune responses (9). However, recent literature clearly established that PRRs such as STING are also involved in self-nucleic acid sensing (10, 11). STING is an endoplasmic reticulum (ER)-associated membrane protein activated by cyclic dinucleotides (CDNs) produced as second messengers by microorganisms or synthesized by enzymes such as cyclic GMP-AMP synthase (cGAS) in response to binding either host- or pathogen-derived cytosolic dsDNA (12, 13). Of note, in addition to cGAS other cytosolic receptors such as DDX41 and IFI16 sense DNA or CDNs to activate STING (12, 13). Activated STING translocates from the ER membrane to the Golgi apparatus, induces nuclear factor κB (NF-κB) and interferon regulatory factor 3 (IRF3) transcription factors activation leading to the production of type I IFNs and other cytokines involved in host immune responses (14). Recent studies showed that STING stimulation can also lead to type III interferon production (15–17). The type III IFN family comprises IFN-λ1 (IL-29), IFN-λ2 (IL-28A), IFN-λ3 (IL-28B), and the newly identified IFN-λ4 in human and IFN-λ2 (IL-28A) and IFN-λ3 (IL-28B) in mice (18). Type III IFN are less well characterized and are thought to function similarly as type I IFN, although in a more restricted manner as their effects are most evident on epithelial cells and neutrophils (18, 19).

Detection of aberrant self-nucleic acids in the cytosol, either mitochondrial DNA (mtDNA) or nuclear DNA (14, 20), is implicated in the development of autoimmune diseases and sterile inflammation (21). Since IPF physiopathology involves unbalanced cell death processes, we investigated whether self-nucleic acid release and its sensing by the STING pathway are participating in the response (1–4). We employed the classical murine model of human IPF using airway exposure to bleomycin (BLM), a potent cytotoxic drug used as chemotherapy but causing pulmonary fibrosis in a fraction of treated patients (22). We show that intranasal BLM administration leads to increased levels of self-DNA in the airways and upregulated cGAS and STING expressions. We report that STING deficiency leads to an exacerbated lung fibrosis independently of type I IFN signaling and characterized by a prolonged neutrophilic inflammation. As a whole, our data show that STING is protective against BLM-induced pulmonary fibrosis in a mechanism that may rely on neutrophilic inflammation resolution.

## Material and Methods

### Mice

Wild-type C57BL/6J (WT) mice were purchased from Janvier laboratory (Janvier Laboratory, France). Mice deficient for STING (*Sting* ^-/-^) were provided by Glen Barber (23), *Cgas* ^-/-^ by Zhijian Chen (24) and *Ifnar1* ^-/-^ by Michel Aguet (25) and bred in our specific pathogen-free animal facility at CNRS (TAAM UPS44, Orleans, France). For experiments, adult (8–14-week-old) males were transferred to experimental animal facility and monitored daily.

### Ethics

All animal experiments complied with the French Government animal experiment regulations and were submitted to the “Ethics Committee for Animal Experimentation of CNRS Campus Orleans” (CCO) under number CLE CCO 2015-1087 and approved under APAFIS#19361. Clinical score was determined daily based on mice appearance and behavior. Appearance was determined based on standard parameters including eye, fur and ear monitoring (https://www.nc3rs.org.uk/grimacescales) and behavior monitoring included mobility, posture and social interaction.

### Treatments

Bleomycin sulfate (7.5 and 3 mg/kg for day 1 and day 14 experiments, respectively; Bellon Laboratories) in saline or saline alone were administered through the airways by nasal instillation in a volume of 40-μl under light isoflurane anesthesia.

### Bronchoalveolar Lavage and Cell Counts

Mice were euthanized by CO_2_ overdose using Prodigy Lab Control Unit (Smartbox) and bronchoalveolar lavage fluid (BALF) was performed as previously described (7). Differential cell counts were routinely performed on cytospin preparation (Cytospin 3, Thermo Shandon) after May-Grünwald Giemsa staining (Sigma-Aldrich, St Louis, MO) according to the manufacturer’s instructions and at least 200 cells were counted using standard morphological criteria.

### Lung Homogenization

After BALF collection, lungs were perfused with isoton to flush the vascular content and homogenized by a rotor-stator (Ultra-turrax^®^) in 1 ml of PBS. The extracts were centrifuged for 10 min at 9,000 g and the supernatants stored at -80°C.

### Lung Histology

Lung left lobes were fixed in 4% buffered formaldehyde, processed and paraffin-embedded under standard conditions. Lung sections (3 μm) were stained with hematoxylin and eosin (H&E) or Sirius red/Fast green. The slides were scanned using NanoZoomer (Hamamatsu Photonics France) and lung fibrosis scored using the Ashcroft modified scale (26).

### Mediator Measurement

BALF supernatants and lung homogenates were analyzed using ELISA assay kits for murine CXCL1/KC, MMP-9, and TIMP-1 according to manufacturer’s instructions (R&D system, Minneapolis, MN). TGF-β1–3 contents in the lungs were assessed by multiplex assay according to manufacturer’s instructions (Merck, Darmstadt, Germany).

### Collagen Assay

BALF collagen content was measured using the Sircol collagen dye binding assay (Biocolor Ltd., Northern Ireland) according to the manufacturer’s instructions.

### Double-Stranded DNA Quantification

Cell-free double-stranded DNA (dsDNA) was measured in the BALF fluid using Quant-iTPicoGreen dsDNA reagent (Invitrogen, Carlsbad, CA), according to the manufacturer’s protocol.

### Quantitative PCR

RNA was purified from lung homogenates using Tri-Reagent (Sigma-Aldrich, Saint-Louis, MO) extraction protocol. RNA reverse transcription into cDNA was carried out with GoTaq qPCR-Master Mix (Promega, Madison, WI). RT-qPCR was performed with Fast SYBR Green Master mix (Promega) on an ARIA MX (Agilent Technologies, Santa Clara, CA). Primers *Tmem173* (#QT00261590) encoding for STING, *Mb21d1* (#QT00131929) encoding for cGAS, and *Fn1* (#QT00135758) encoding for fibronectin were purchased from Qiagen (Qiagen, Hilden, Germany). RNA expression was normalized to *Gapdh* (#QT00166768, Qiagen, Hilden, Germany) expression and analyzed using the ΔΔCt method.

### Flow Cytometry

Lungs were cut in small pieces and digested using a 1mg/ml DNase (DN25, Sigma-Aldrich, St Louis, MO)/125 µg/ml collagenase (Liberase™, Sigma-Aldrich, St Louis, MO) solution for 45 min at 37°C and filtered on 40 µm cell strainer. BALF and lung single cell suspensions were incubated for 20 min at RT with Fc block (Thermo Fisher Scientific, Waltham, MA), washed and stained with surface markers including a Fixable Viability Dye eFluor 780 (Thermo Fisher Scientific, Waltham, MA). Cells were fixed and permeabilized using Fixation/Permeabilization kit (Thermo Fisher Scientific, Waltham, MA) and stained with intracellular markers. For IL-17 staining (clone eBio17B7), lung cell suspensions were incubated for 5 h at 37°C in presence of brefeldin A (BFA; Thermo Fisher Scientific, Waltham, MA) to prevent protein secretion. Antibody clones and dilutions used are detailed in **Supplementary Table 1**. All samples were acquired on an LSR Fortessa flow cytometer (BD Biosciences, San Jose, CA) and analyzed using FlowJo software (TreeStar).

### Immunoblot

20μg of proteins (Pierce BCA protein assay, Thermo Fisher Scientific, Waltham, MA) were denatured by boiling (95°C, 5 min) in reducing SDS sample buffer, separated by SDS-PAGE and transferred to nitrocellulose membranes (GE Healthcare Life Sciences, Amersham, UK). After blocking in 5% Blotting-Grade Blocker (BioRad, France) and washing in Tris-Buffered saline (TBS)- 0,1% Tween^®^ 20, membranes were incubated with primary mouse anti-STING (Abcam, ref ab92605), anti-cGAS (Cell Signaling Technology, ref 3165) or anti-IL-28 (Santa Cruz Biotechnology, ref sc-137151) antibodies in TBS-BSA (Bovine Serum Albumine) 1%- azide 0,5 mM overnight at 4°C. Membranes were washed and incubated with relevant secondary antibody conjugated to horseradish peroxidase (HRP) 2 h at RT. Anti-actin antibody was already HRP-conjugated (Sigma-Aldrich, ref A3854). Detection was performed with ECL Western-blotting Detection Reagent (GE Healthcare Life Sciences, Amersham, UK) and luminescence acquired using Multi-application gel imaging system PXi software (Syngene). Bands intensity were quantified using ImageJ (NIH, USA).

### Immunofluorescence

Lung tissues were fixed in 4% paraformaldehyde (PFA) (Sigma-Aldrich, St Louis, MO) and then dehydrated in 30% sucrose solution for 2 weeks at 4°C. Lungs were embedded in tissue-teck (OCT^®^) and stored at -80°C. 10-μm lung sections were cut on cryostat (Leica, Solms, Germany) and heated at 80°C for 30 min in 10 mM citrate pH = 6. Lung cells were permeabilized with 0.5% Triton X-100, blocked in 1% BSA 10% SVF 0.1% Triton X-100 in PBS for 1 h, washed three times in TBS, and incubated overnight with rabbit anti-STING (ab92605 1/50, Abcam) in PBS containing 2% BSA, 10% FCS, and 0.5% Triton X-100 at 4°C. Lung sections were TBS washed and incubated with anti-rabbit IgG Alexa 532 secondary antibody (1/100) for 1 h at RT. After washing, cells were counterstained with DAPI for 10 min at RT, PBS washed, and mounted onto microscope slides (mowiol). Slides were observed using a Zeiss Axiovert 200M microscope coupled with a Zeiss LSM 510 Meta scanning device (Carl Zeiss Co. Ltd., Jena, Germany). The inverted microscope was equipped with a Plan-Apochromat 63X objective (NA = 1.4). Images were acquired using Zeiss LSM Image Browser (Carl Zeiss Co. Ltd., Jena, Germany).

### Statistical Analyses

Statistical tests of selected populations were performed using Mann-Whitney non-parametric test. Weight variation plots were analyzed using two-way ANOVA corrected for multiple comparisons employing Tuckey’s test. Results were considered significant at p < 0.05.

## Results

### BLM Administration Induces Airway Self-DNA Release and Lung cGAS and STING Expressions

**Figure 1:**
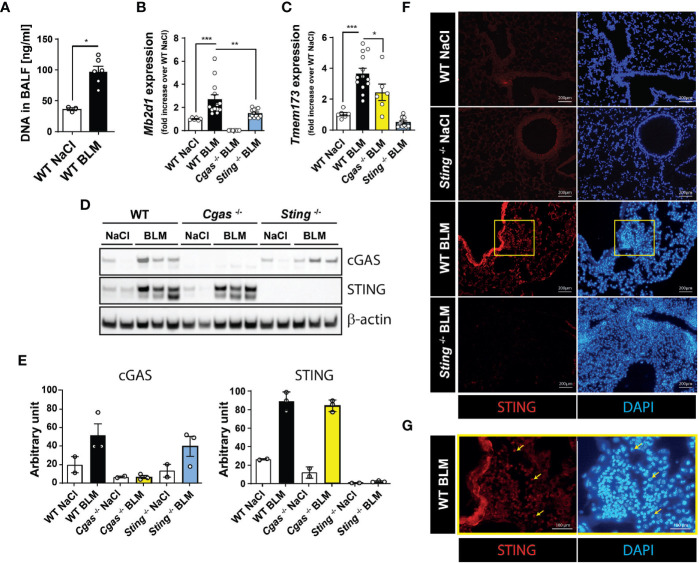
Increased BALF dsDNA content as well as cGAS and STING expressions. C57BL/6 WT, *Cgas* ^-/-^ and *Sting* ^-/-^ mice were treated with NaCl or BLM (3 mg/kg intranasally) and BALF and lungs were collected after 14 days. **(A)** BALF cell-free dsDNA content. **(B)** *Mb21d1* (cGAS) and **(C)** *Tmem173* (STING) gene expressions. STING protein expression by **(D)** Western-Blotting and **(E)** semi-quantitative band analysis. **(F)** STING expression in the lungs assessed by immunofluorescence. The yellow box is magnified in **(G)** and yellow arrows illustrate STING positive infiltrating cells. Data are representative of 2-3 independent experiments, showed as mean ± SEM, *p < 0.05; ***p < 0.001.

To address a potential role for self-DNA recognition by the cGAS/STING pathway in the establishment of pulmonary fibrosis, we measured double stranded (ds) DNA content in the bronchoalveolar lavage fluid (BALF) as well as cGAS and STING expressions in the lungs at the fibrotic phase (14 days after BLM instillation). As compared to saline (NaCl) control wild-type (WT) mice, we noted that dsDNA content is significantly increased in the BALF of BLM-treated WT mice (**Figure 1A**) and BLM treatment leads to a strong increase of cGAS (*Mb21d1*) (**Figure 1B**) and STING (*Tmem173*) (**Figure 1C**) gene expressions in the lungs. Of note, *Mb21d1* and *Tmem173* expressions are reduced in absence of STING and cGAS, respectively, suggesting retroactive loops at the gene expression level. We next assessed cGAS and STING protein expressions in saline- or BLM-treated WT mice, cGAS (*Cgas* ^-/-^) or STING (*Sting* ^-/-^) deficient mice. Both cGAS and STING protein expressions are increased in the lungs of BLM-treated WT mice and cGAS or STING levels are not impaired by the absence of STING or cGAS, respectively (**Figures 1D, E**). We also assessed STING expression in pulmonary tissue by immunofluorescence and show that it is expressed in both bronchial epithelial cells and infiltrating cells (**Figures 1F, G**).

Regarding the acute inflammatory phase (1 day after BLM instillation), we observed that despite a strong increase of BALF dsDNA content (**Supplementary Figure 1A**), cGAS or STING deficiency had no major impact on total BALF cell numbers (**Supplementary Figure 1B**) or neutrophil frequencies (**Supplementary Figure 1C**). Lung levels of the neutrophil attractant chemokine CXCL1 as well as the remodeling factors MMP-9 and TIMP-1 (**Supplementary Figures 1D–F**) were also unchanged among BLM-treated groups suggesting minor cGAS/STING contribution in early inflammation. Nevertheless, persistent enhanced airway dsDNA in the BALF associated with a strong increase of lung cGAS and STING expressions at the fibrotic stage led us to investigate a role for this pathway in the establishment of pulmonary fibrosis.

### STING Deficiency Leads to Increased Pulmonary Fibrosis

**Figure 2:**
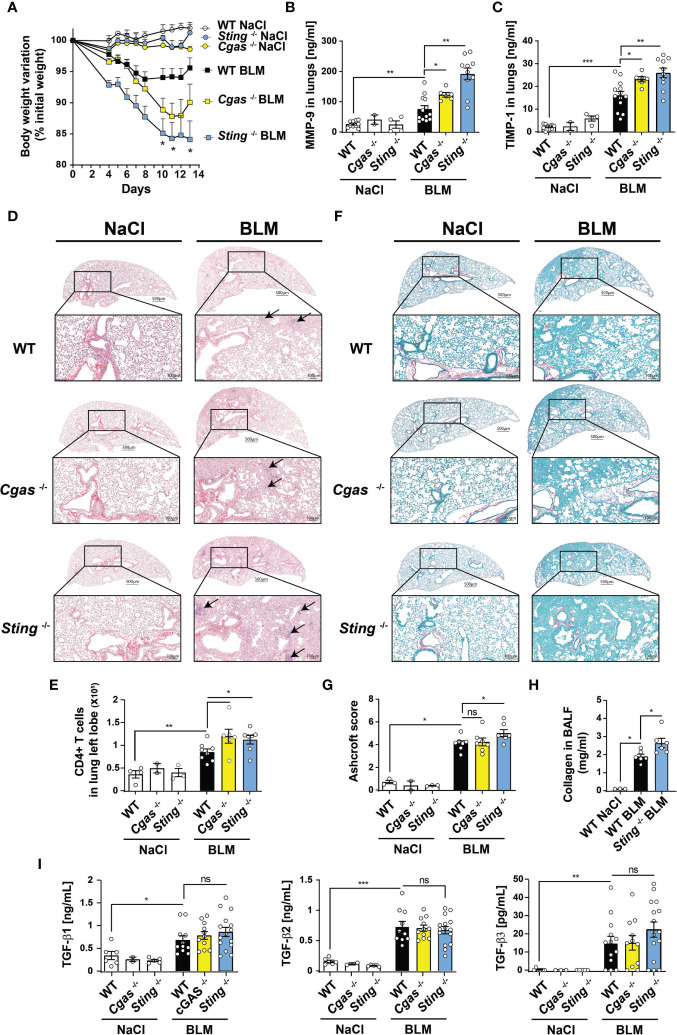
Increased fibrosis in absence of STING. WT, *Cgas* ^-/-^ and *Sting* ^-/-^ mice were treated with NaCl or BLM (3 mg/kg intranasally) and BALF and lungs were collected after 14 days. **(A)** Body weight evolution. Asterisks depict p < 0.05 comparing BLM-treated WT and *Sting* ^-/-^ groups at indicated time points **(B)** MMP-9 and **(C)** TIMP-1 levels in the lungs measured by ELISA. **(D)** Lung micrographs stained by Hematoxylin and Eosin (H&E). **(E)** CD4^+^ T lymphocyte numbers in the lung left lobe. **(F)** Lung micrographs stained by Red Sirius/Fast Green highlighting collagen fibers. **(G)** Fibrosis score using Ashcroft modified scale. **(H)** BALF collagen content measured by Sircol assay. **(I)** TGF-β1-3 in the lungs measured by multiplex assay. Data are representative of 2-3 independent experiments, showed as mean ± SEM, ns: non-significant; *p < 0.05; **p < 0.01; ***p < 0.001.

As compared to BLM-treated WT or *Cgas* ^-/-^ mice, *Sting* ^-/-^ mice exhibit higher body weight loss (**Figure 2A**) with occasional succumbing animals. The remodeling factors MMP-9 (**Figure 2B**) and TIMP-1 (**Figure 2C**) were also increased in the lungs of STING deficient animals as compared to their WT relatives 14 days post BLM administration. This was accompanied by higher lung cell accumulation as shown by H&E staining of lung micrographs (**Figure 2D**) and CD4^+^ T lymphocyte numbers assessed by flow cytometry (**Figure 2E**). Sirius Red/Fast Green collagen staining also shows extended areas of fibrosis and increased collagen deposition (**Figure 2F**), further confirmed by fibrosis score using Ashcroft modified scale (26) (**Figure 2G**). BALF collagen content was also increased in *Sting* ^-/-^ mice as compared to their WT relatives (**Figure 2H**). Interestingly, BLM-treated *Cgas* ^-/-^ mice display intermediate bodyweight loss (**Figure 2A**), MMP-9 production (**Figure 2B**) and fibrosis (**Figures 2F, G**), suggesting that other DNA sensors might be involved. Of note, BLM-induced increased levels of the important pro-fibrotic factor TGF-β were not significantly altered in cGAS or STING deficient mice (**Figure 2I**), arguing for a role of another pro-fibrotic pathway in absence of STING.

### STING-Mediated Protection Is Type I IFN Signaling Independent

**Figure 3:**
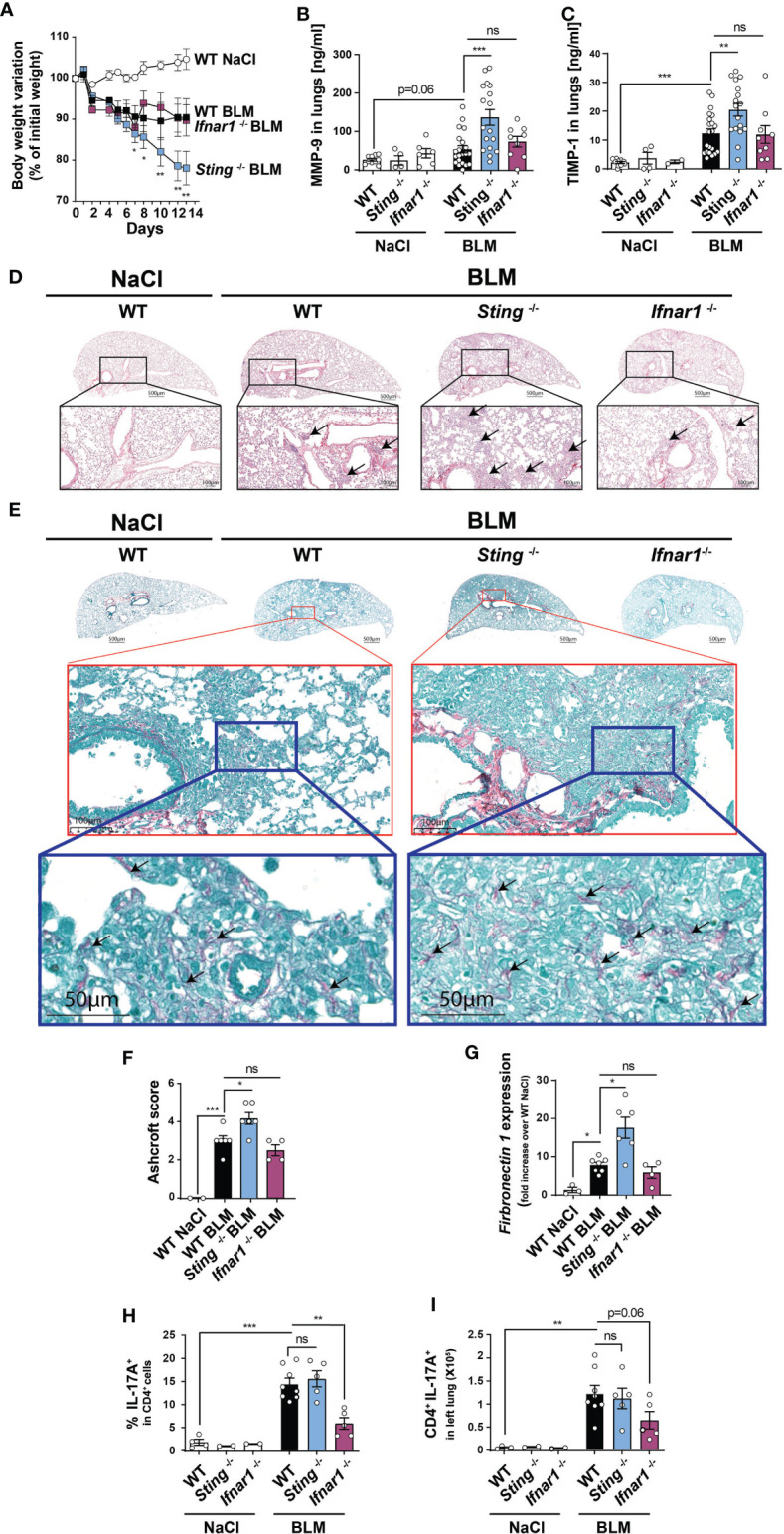
STING deficiency leads to increased fibrosis independently of type I IFN signaling. WT, *Sting* ^-/-^, and type I Interferon receptor deficient (*Ifnar1* ^-/-^) mice were treated with NaCl or BLM (3 mg/kg intranasally), and BALF and lungs were collected after 14 days. **(A)** Body weight evolution. Asterisks depict p < 0.05 comparing BLM-treated WT and *Sting* ^-/-^ groups. **(B)** MMP-9 and **(C)** TIMP-1 levels in the lungs measured by ELISA. Lung micrographs stained by **(D)** Hematoxylin and Eosin (H&E) (black arrows show cell foci) and **(E)** Red Sirius/Fast Green highlighting collagen fibers. Higher magnification are depicted using red and blue rectangles and black arrows show collagen deposition. **(F)** Fibrosis score using Ashcroft modified scale. **(G)** Lung *fibronectin 1* gene expression by RT qPCR. **(H)** Frequencies and **(I)** total numbers of lung CD4^+^ IL-17^+^ T cells assessed by flow cytometry. Data are representative of 2-3 independent experiments, showed as mean ± SEM, ns: non-significant *p < 0.05; **p < 0.01; ***p < 0.001.

STING signaling pathway is a well-characterized type I IFN inducer (14). We thus utilized mouse deficient for type I IFN receptor (*Ifnar1* ^-/-^) to test the hypothesis of a type I IFN-dependent role of STING. In contrast to BLM-treated *Sting* ^-/-^ mice, *Ifnar1* ^-/-^ displayed comparable body weight loss as WT mice (**Figure 3A**). In addition, lung remodeling factors MMP-9 and TIMP-1 were similarly increased comparing BLM-treated WT versus *Ifnar1* ^-/-^ mice, while *Sting* ^-/-^ mice displayed higher expressions (**Figures 3B, C**). *Ifnar1* ^-/-^ mice did not display enhanced lung cell influx (**Figure 3D**) and lung fibrosis as shown by representative lung histology (**Figure 3E**) and Ascroft fibrosis score (**Figure 3F**) and the expression of the extracellular matrix protein fibronectin (*Fn1*) (**Figure 3G**). Together, these results suggest that STING is protective against BLM-induced fibrosis in a type I IFN-independent manner. Since IL-17A is a major pro-fibrotic cytokine (27) and in line with a recent study showing that STING-mediated protection relies of IL-17A modulation (28), we analyzed IL-17A production by CD4^+^ T lymphocytes using flow cytometry (**Supplementary Figures 2A, B**). We show no difference in terms of frequencies (**Figure 3H**) or total numbers (**Figure 3I**) of IL-17^+^ CD4^+^ T cells comparing WT and *Sting* ^-/-^ mice. Unexpectedly, *Ifnar1* ^-/-^ CD4^+^ T lymphocytes displayed lower frequency of IL-17+ cells in the lungs. Having excluded a major role for type I IFN and IL-17A to explain STING-dependent protection, we next investigated a potential role for IL-28 (type III IFN) (15–17). Interestingly, we show that BLM induces IL-28 in the lungs and that its production partially depends on STING (**Supplementary Figures 3A–C**).

### STING Deficiency Affects Neutrophil Numbers and Function

**Figure 4:**
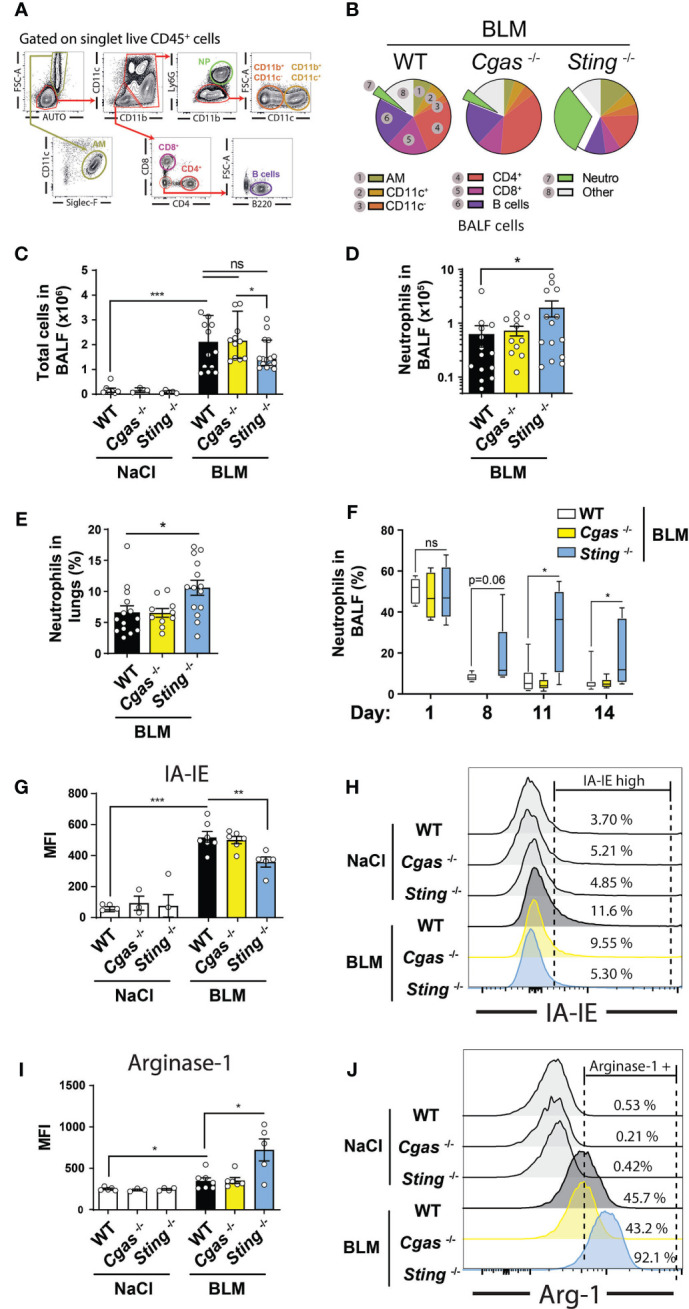
STING deficiency leads to dysregulated neutrophils. WT, *Cgas* ^-/-^ and *Sting* ^-/-^ mice were treated with NaCl or BLM (3 mg/kg intranasally) and BALF and lungs were collected after 14 days. **(A)** Flow cytometry gating strategy used to identify **(B)** BALF cell populations. Cells are gated on singlets, live, CD45^+^ events. NP: Neutrophils; AM: Alveolar macrophages. Total BALF cell numbers **(C)**, neutrophil **(D)** BALF total numbers and **(E)** lung percentages. **(F)** Kinetic of BALF neutrophil frequencies at indicated time points. MHC-II (I-A/I-E) **(G, H)** and arginase-1 (Arg-1) **(I, J)** Mean Fluorescence Intensity (MFI) assessed by flow cytometry. Data are representative of two to three independent experiments, showed as mean ± SEM, *p < 0.05; ***p < 0.001.

The fibrotic stage of the pathology is accompanied by the recruitment of adaptive immune cells. As expected, at this time point the main BALF cell populations in WT mice are CD4^+^ and CD8^+^ T lymphocytes as well as B lymphocytes, and the proportion of neutrophils is low (**Figures 4A, B**). In contrast, neutrophils remained high in BALF and lungs of STING-deficient mice (**Figures 4B–E** and **Supplementary Figures 4A, B**). We performed kinetic studies and found that BALF neutrophils persisted by days 8–14 post-BLM treatment (**Figure 4F**), suggesting prolonged inflammation. In addition to increased numbers in the BALF, lung STING-deficient neutrophils (**Supplementary Figure 4B**) display reduced MHC-II (I-A/I-E) upregulation following BLM treatment as compared to their WT counterparts (**Figures 4G, H**). In contrast, STING-deficient neutrophils show a strong upregulation of arginase-1 expression (**Figures 4I, J**), suggesting that STING controls neutrophil persistence and function.

## Discussion

STING has been identified in 2008 as an endoplasmic reticulum receptor that induces innate immune responses (29) and is currently a hot topic in several fields including cancer immunotherapy (30), vaccines (31) and autoimmunity (32, 33). Depending on the microenvironment and responsive cell types, enhancing STING signaling pathway might favor antitumor activity for instance by promoting IFN-β-dependent T cell priming (34). On the other hand, constitutive self-DNA-mediated sustained STING activation induces tolerance breakdown and autoimmunity (32, 33).

There are several STING allelic variants in the general population (35) and STING-associated vasculopathy with onset in infancy (SAVI) is an autoinflammatory disease caused by gain-of-function mutations in *TMEM173* (36, 37). Affected children display constitutive STING activation leading to increased *IFNB1* transcription and even higher transcripts levels upon cGAMP stimulation, whereas other pro-inflammatory gene levels such as interleukin-1 (*IL1*), interleukin-6 (*IL6*), and tumor necrosis factor (*TNF*) remain unaffected (36). In addition to cutaneous vasculopathy, a major feature of SAVI is interstitial lung disease (38). Of note, the STING knock-in mouse strain (V154M) corresponding to a recurrent mutation in SAVI patients exhibit a severe combined immunodeficiency disease (SCID) phenotype (39).

Here, we employed the classical murine model of idiopathic pulmonary fibrosis (IPF) induced by BLM administration in the airways. We show that BLM induces a strong increase of cell-free self-DNA in the airways, which can act as a danger signal by triggering DNA sensing pathways to activate innate immune responses (40) and notably in the lungs (11). However, the mechanisms by which self-DNA becomes accessible for intracellular DNA sensors remain uncertain. Several context-dependent pathways have been reported, such as IgG- or HMGB1-bound DNA internalization following interaction with FcγRIIa or receptor for advanced glycation end products (RAGE), respectively (41). The antimicrobial peptide LL37 was shown to transport extracellular DNA into the cytoplasm of human primary monocytes triggering STING activation (42). IL-10-family member IL-26 binds to genomic, mtDNA or neutrophil extracellular traps (NETS) DNA and traffic them into the cytosol of human myeloid cells activating STING (43). Further investigations are required to decipher the respective contributions of nuclear DNA versus mtDNA in activating the STING pathway. Elevated plasma mtDNA copy numbers in IPF patients predicts death (44); however, it remains unsure whether mtDNA is a mere marker or whether it actually modulates pathology.

Investigating sensors involved in self-DNA recognition, our results indicate that the cGAS/STING pathway does not seem to play a major role in the early airway inflammatory response, as we did not observe major changes in terms of canonical markers such as neutrophil recruitment and remodeling factors production comparing WT versus cGAS or STING deficient mice. In contrast, our data show that STING-dependent responses are important during the fibrotic stage of the disease. First, lung cGAS and STING expressions are increased both at gene and protein levels. Interestingly, Mb21d1 (encoding for cGAS) gene expression but not cGAS protein is decreased in STING deficient animals suggesting potential cross regulation. In addition, STING deficiency leads to increased lung fibrosis, as characterized by higher histological score, collagen deposition and remodeling factors expression. A protective role of STING in IPF patients has been reported recently. Authors show that blood mononuclear cells from patients undergoing acute exacerbation display significantly reduced STING protein levels correlating with decreased partial oxygen pressure (45). In addition, patients showing clinical improvement post-treatment had higher STING protein levels as compared to patients with worsen condition (45). STING has also been shown to elicit protective responses in experimental autoimmune encephalitis by dampening effector T cell infiltration and inducing dominant T cell regulatory response (46). On the other hand, STING contributes to pathology in a number of other disease settings, including silica-induced lung inflammation (47) and carbon-tetrachloride (CCl4)-induced liver fibrosis (48). These discrepancies might reflect fine-tuning of STING signaling as well as potential differences in the kinetics and cell subsets involved.

We sought to determine STING-dependent signaling pathway mediating protection in the context of lung fibrosis and first addressed the role of type I IFN. Interestingly, our results show that STING-dependent effect does not rely on type I IFN signaling as mice deficient for its receptor do not display exacerbated pathology. While type I IFN induction is a major consequence of STING activation, several publications demonstrated that this cytokine does not directly contribute to STING-dependent effect in a number of situations. For instance, STING-associated lung disease due to a gain of function mutation in the protein (N153S) is T cell dependent but does not require IRF3/IRF7 or IFNAR (49). Similarly, SCID phenotype in STING V154M mice occurs similarly when type I IFN signaling is absent (39). STING-associated vasculopathy also develops independently of IRF3 in mice (50). Besides type I IFN induction, several publications showed STING-dependent type III IFN induction (IL-28/IL-29) in the context of viral infection (16) and exogenous DNA (17) or di-GMP mucosal adjuvant (15) stimulations. Interestingly, a SAVI patient presented increased plasma IL-29 expression (51). Here, we show that BLM triggers IL-28 and that its production is decreased in STING-deficient mice as compared to their WT relatives. Type III IFN was shown to suppress neutrophil response either by directly limiting its influx in a collagen-induced arthritis model (52) or by primarily modifying neutrophil effector functions *via* posttranslational modifications in the gut (53). Follow-up experiments are needed to delineate the exact contribution of type III interferons in experimental lung fibrosis. Another possibility to explain a protective role of STING was its potential action on IL-17A production, a potent pro-fibrotic cytokine (27). Indeed, STING deficiency was reported to enhance Th17 polarization and IL-17A production therefore promoted pancreatic inflammation and fibrosis (28). However, in our model IL-17A production by CD4^+^ T cells was not increased in STING deficient mice as compared to their WT relatives. In our experimental setup, we found that while mice deficient for type I IFN signaling did not show altered fibrosis, their lung CD4^+^ T lymphocytes display lower frequencies of IL-17A^+^ cells. In addition, our data indicate that cGAS deficiency does not fully mirror STING deficiency phenotype, as BLM-treated *Cgas* ^-/-^ mice displayed increased expression of the fibrosis-associated remodeling factors MMP-9 and TIMP-1 as compared to WT animals but intermediate weight loss and similar Ashcroft histological score. These results suggest an intricate role of cGAS in the development of experimental lung fibrosis and that other DNA sensors might be involved such as DDX41 or IFI16 (13, 54, 55). Interestingly, it was recently shown that etoposide-induced DNA damage leads to cGAS-independent STING activation (56), while on the other hand cGAS protects hepatocytes by triggering autophagy independently of STING in mouse models of ischemia-reperfusion (57).

We report that STING deficiency leads to sustained BALF and lung neutrophil infiltration following BLM administration. Additional experiments are required to determine whether prolonged BALF and lungs neutrophil presence in the context of STING deficiency reflects different tissue lifespan and/or recurrent neutrophils recruitment. Airway neutrophilia has been associated with early mortality in IPF (58) and concentrations of the neutrophil chemoattractant CXCL8 are increased in IPF patients (59). Interestingly, we show that lung neutrophils from STING deficient mice exhibit higher levels of arginase-1. L-arginine catabolism by iNOS and arginase is related to cytotoxicity and tissue repair and classically associated with pro- versus anti-inflammatory functions of macrophages, respectively (60, 61). Interestingly, L-arginine levels in the lungs of BLM-treated mice decrease due to increased arginase-1 expression and the addition of an arginase inhibitor limits TGF-β-induced collagen production by fibroblasts (62). It remains to be investigated whether neutrophil-derived arginase-1 can influence fibroblast function. In addition, we also report lower MHC II levels on STING-deficient lung neutrophils. Neutrophil are able to perform MHC-II-mediated antigen presentation to CD4^+^ T cells (63) and recent literature reviewed regulatory roles of neutrophils in adaptive immunity (64). However, the exact meaning of reduced MHC-II expression on STING-deficient neutrophils following BLM treatment is unclear, potentially reflecting altered T cell activation properties. As a whole, our data show an unexpected regulatory function of STING to limit BLM-induced pulmonary fibrosis associated with neutrophilic inflammation regulation.

## Data Availability Statement

The original contributions presented in the study are included in the article/**Supplementary Material**. Further inquiries can be directed to the corresponding authors.

## Ethics Statement

The animal study was reviewed and approved by Ethics Committee for Animal Experimentation of CNRS Campus Orleans (CCO) 2015-1087 and APAFIS#19361.

## Author Contributions

FS, CS, NL-Q, MM, MN, SH-M, FG, AG, and NR performed experiments and/or analyzed data. NR and IC conceived and directed the project with assistance from AG, MB, and BR. NR and IC wrote the manuscript. All authors had the opportunity to discuss the results and comment on the manuscript. All authors contributed to the article and approved the submitted version.

## Funding

This work was supported by Centre National de la Recherche Scientifique (CNRS), the University of Orleans, The Region Centre Val de Loire (2003–00085470), the «Conseil Général du Loiret» and the European Regional Development Fund (FEDER no. 2016-00110366 and EX005756).

## Conflict of Interest

The authors declare that the research was conducted in the absence of any commercial or financial relationships that could be construed as a potential conflict of interest.

## Supplementary Material

Supplementary Figure 1 WT, *Cgas* ^-/-^ and *Sting* ^-/-^ mice were treated with NaCl or BLM (7 mg/kg intranasally) and BALF and lungs were collected at 24 h. **(A)** BALF cell-free dsDNA content. BALF **(B)** total numbers and **(C)** neutrophil frequencies. KC/CXCL-1 **(D)**, MMP-9 **(E)** and TIMP-1 **(F)** levels in the lungs measured by ELISA. Data are a pool of two to three independent experiments showed as mean ± SEM, ns: non-significant; ***p < 0.001.

Supplementary Figure 2 WT, *Sting* ^-/-^ and *Ifnar1* ^-/-^ mice were treated with NaCl or BLM (3 mg/kg intranasally) and lungs cells were collected after 14 days and incubated for 5 h at 37°C in presence of BFA. **(A)** Gating strategy employed to delineate lung IL-17A^+^ CD4^+^ T lymphocytes. **(B)** Representative flow cytometry plots. Data are representative of 2 independent experiments.

Supplementary Figure 3 WT, *Cgas* ^-/-^, *Sting* ^-/-^ and *Ifnar1* ^-/-^ mice were treated with NaCl or BLM (3 mg/kg intranasally) and lungs were collected after 14 days. **(A)** IL-28 content in the lungs measured by ELISA and **(B)** by Western-Blotting with **(C)** semi-quantitative band analysis. Data are representative of 2 independent experiments showed as mean ± SEM, ns: non-significant; *p < 0.05

Supplementary Figure 4 Gating strategy used to delineate neutrophil populations **(A)** in the BALF and **(B)** in the lungs by flow cytometry.
