# Supplementary figures and images for: Protective Role of the Nucleic Acid Sensor STING in Pulmonary Fibrosis
Source: Front Immunol. 2021 Jan 8;11:588799. doi: 10.3389/fimmu.2020.588799 (PMC7820752; doi:10.3389/fimmu.2020.588799)

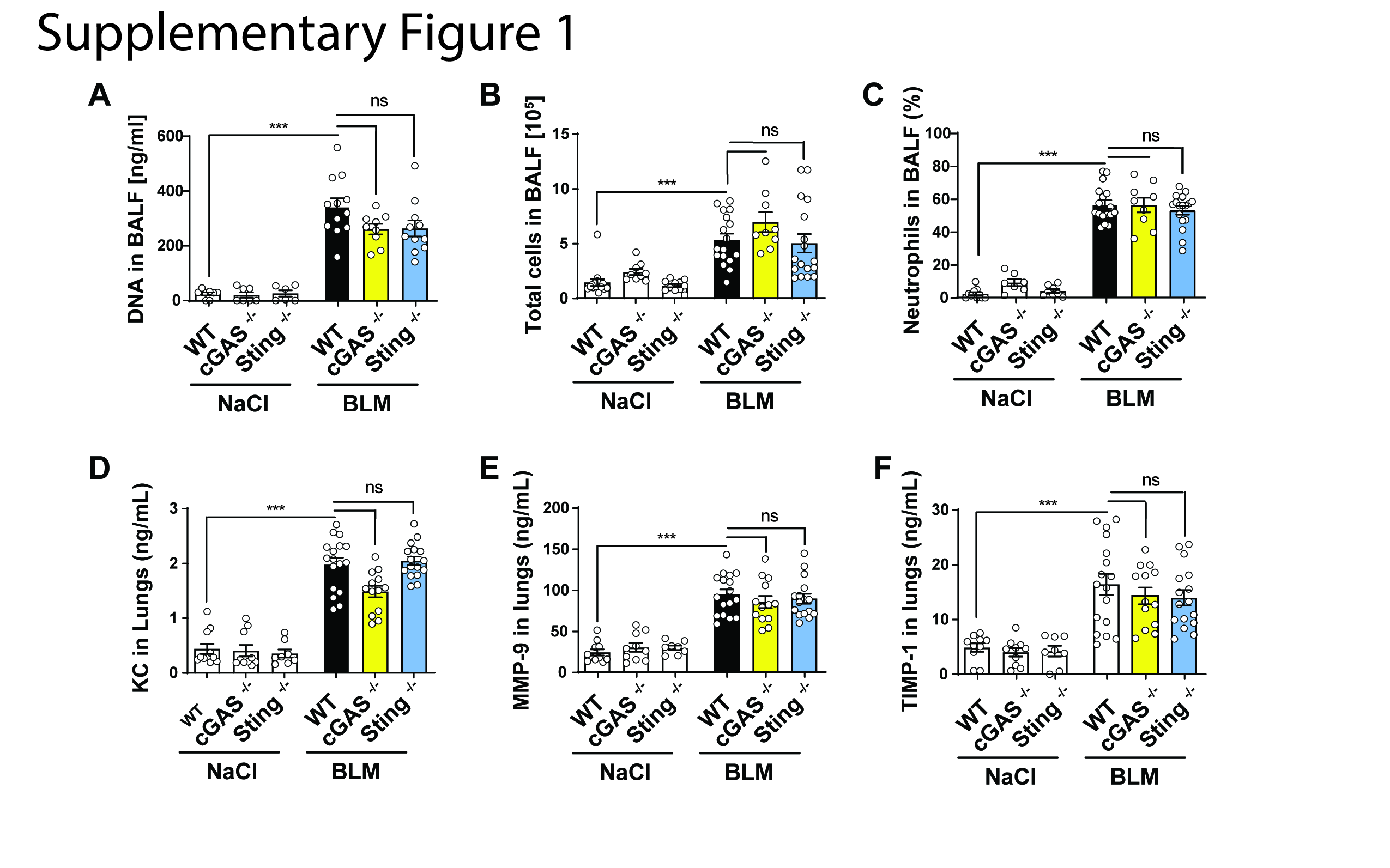

Supplement: Supplementary Figure 1 — WT, Cgas -/- and Sting -/- mice were treated with NaCl or BLM (7 mg/kg intranasally) and BALF and lungs were collected at 24 h. (A) BALF cell-free dsDNA content. BALF (B) total numbers and (C) neutrophil frequencies. KC/CXCL-1 (D), MMP-9 (E) and TIMP-1 (F) levels in the lungs measured by ELISA. Data are a pool of two to three independent experiments showed as mean ± SEM, ns: non-significant; ***p < 0.001. [file Image_1.tif]

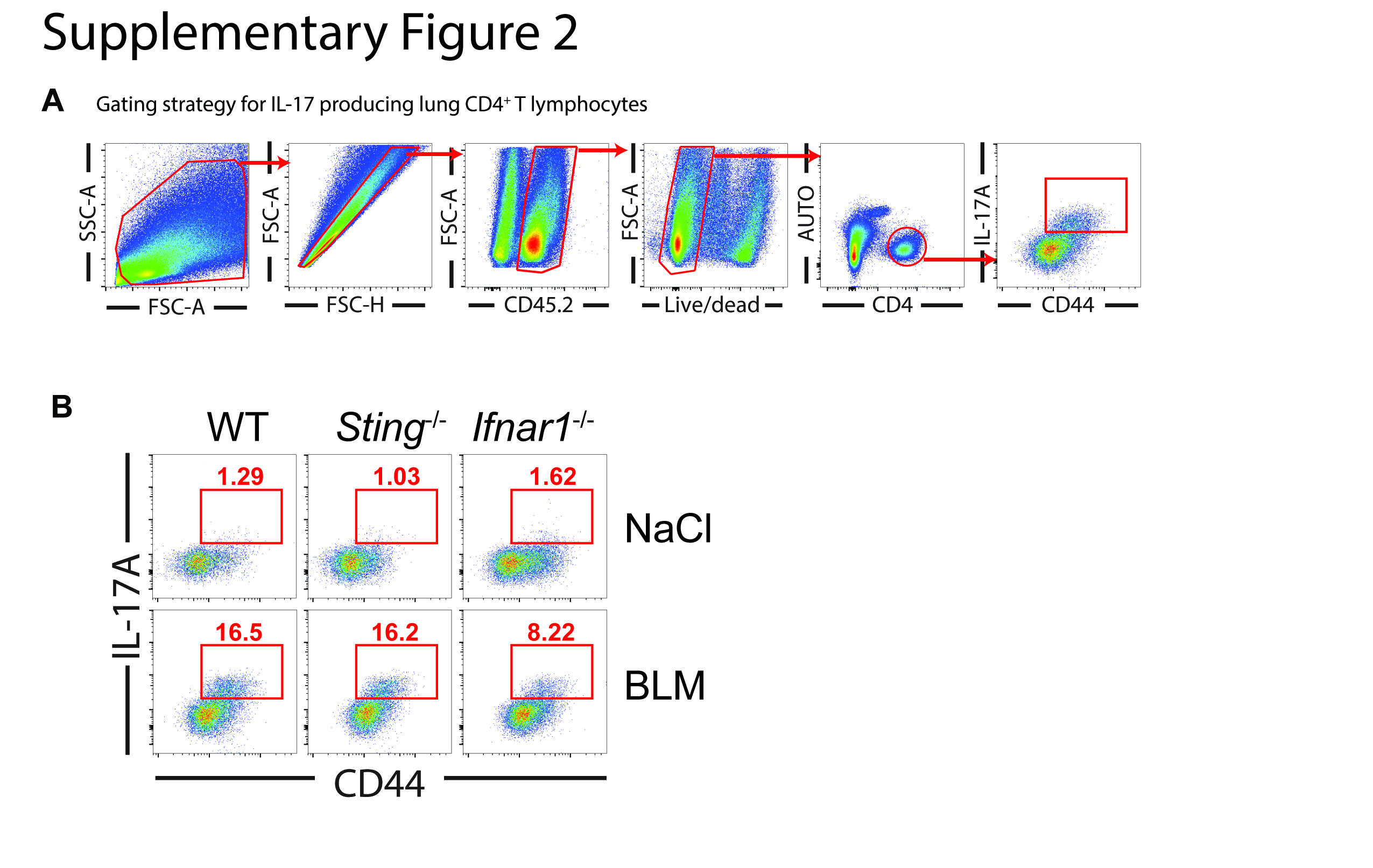

Supplement: Supplementary Figure 2 — WT, Sting -/- and Ifnar1 -/- mice were treated with NaCl or BLM (3 mg/kg intranasally) and lungs cells were collected after 14 days and incubated for 5 h at 37°C in presence of BFA. (A) Gating strategy employed to delineate lung IL-17A+ CD4+ T lymphocytes. (B) Representative flow cytometry plots. Data are representative of 2 independent experiments. [file Image_2.tif]

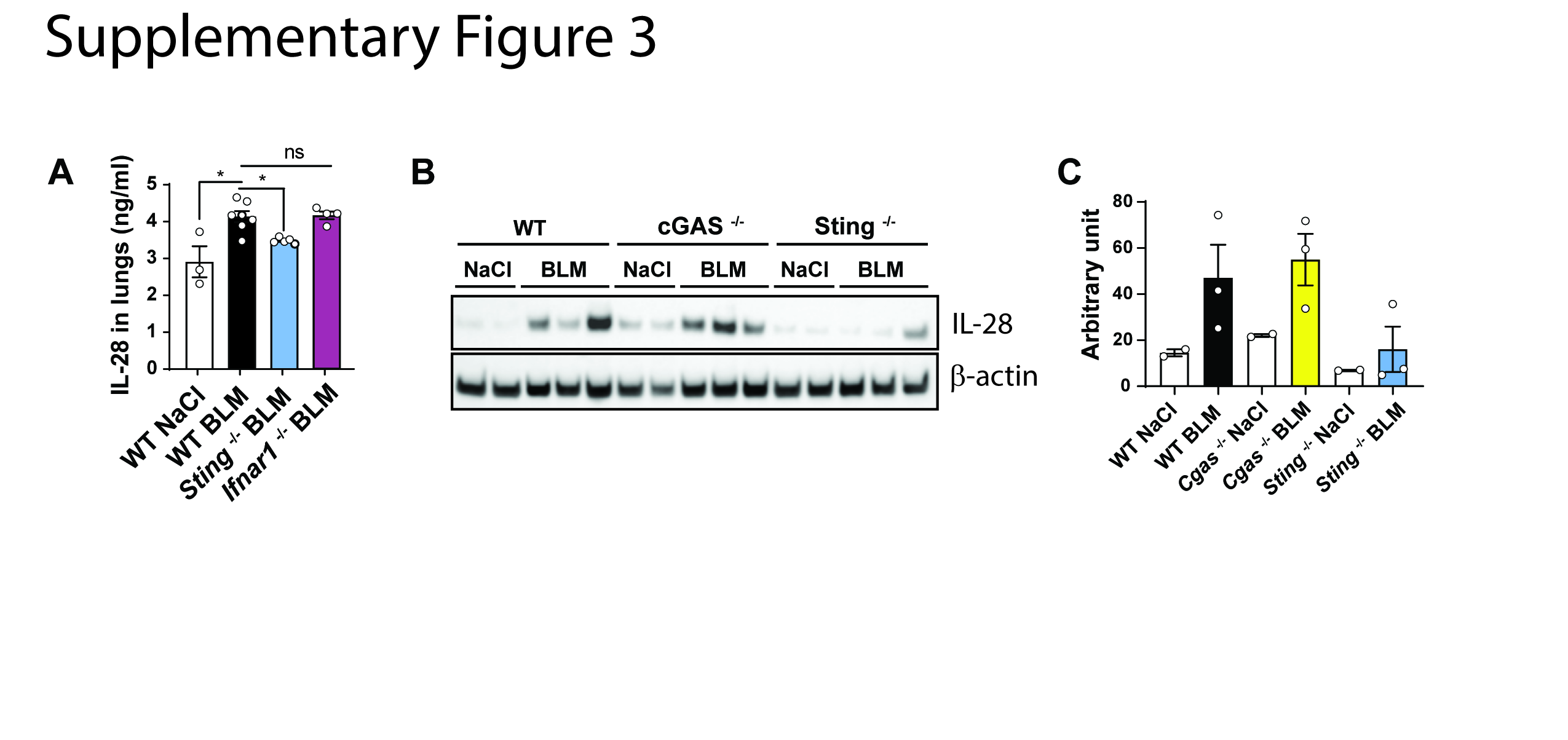

Supplement: Supplementary Figure 3 — WT, Cgas -/-, Sting -/- and Ifnar1 -/- mice were treated with NaCl or BLM (3 mg/kg intranasally) and lungs were collected after 14 days. (A) IL-28 content in the lungs measured by ELISA and (B) by Western-Blotting with (C) semi-quantitative band analysis. Data are representative of 2 independent experiments showed as mean ± SEM, ns: non-significant; *p < 0.05 [file Image_3.tif]

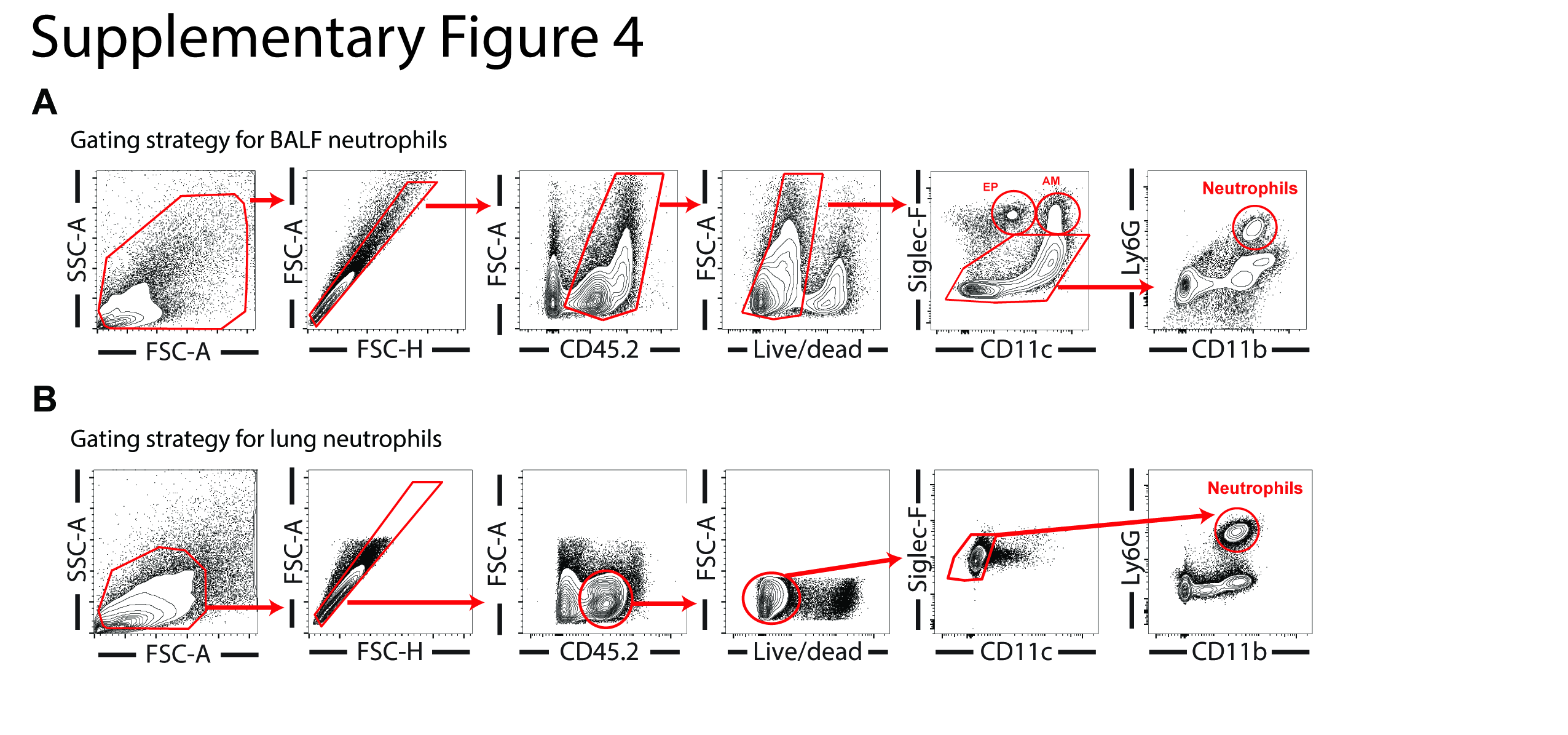

Supplement: Supplementary Figure 4 — Gating strategy used to delineate neutrophil populations (A) in the BALF and (B) in the lungs by flow cytometry. [file Image_4.tif]

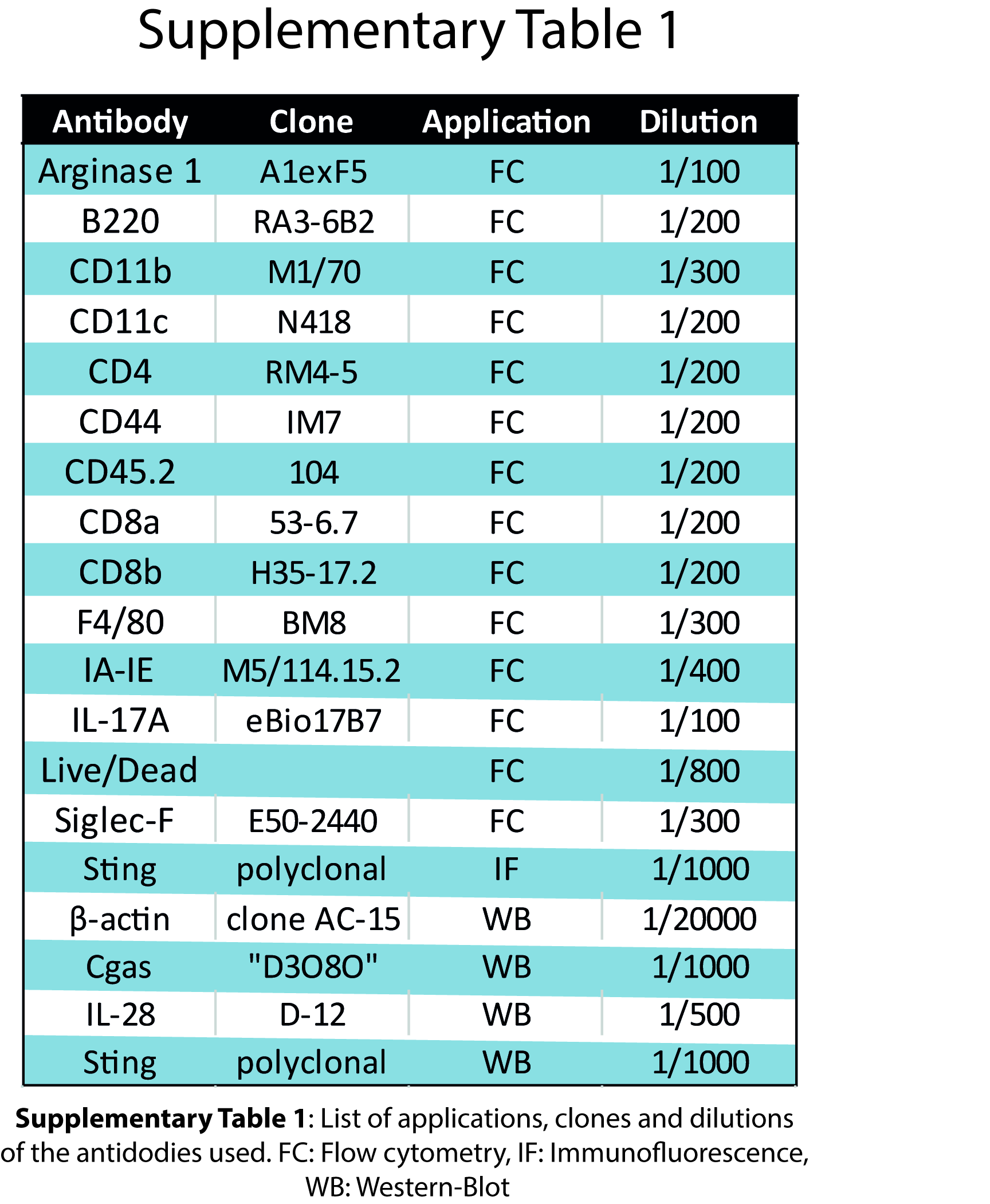

Supplement: Supplementary file 5 [file DataSheet_1.zip › Supplementary Table 1.TIF]
